# The Potential Influence of the Presence of Mycotoxins in Human Follicular Fluid on Reproductive Outcomes

**DOI:** 10.3390/toxins16120509

**Published:** 2024-11-25

**Authors:** Apolka Szentirmay, Zsófia Molnár, Patrik Plank, Miklós Mézes, Attila Sajgó, Attila Martonos, Tímea Buzder, Miklós Sipos, Lili Hruby, Zsuzsanna Szőke, Levente Sára

**Affiliations:** 1 Department of Obstetrics and Gynecology, Semmelweis University, 1088 Budapest, Hungary; szentirmay.apolka@gmail.com; 2 Department of Animal Biotechnology, Agribiotechnology and Precision Breeding for Food Security National Laboratory, Institute of Genetics and Biotechnology, Hungarian University of Agriculture and Life Sciences, 2100 Gödöllő, Hungary; molnar.zsofia@uni-mate.hu (Z.M.); plankpatrik94@gmail.com (P.P.); 3 Department of Feed Safety, Institute of Physiology and Nutrition, Hungarian University of Agriculture and Life Sciences, 2100 Gödöllő, Hungary; mezes.miklos@uni-mate.hu; 4 Central of Assisted Reproduction, Semmelweis University, 1097 Budapest, Hungary; sajgo.attila@semmelweis.hu (A.S.); buzder.timea@semmelweis.hu (T.B.); sipos.miklos@semmelweis.hu (M.S.); 5 Faculty of Medicine, University of Freiburg, 79110 Freiburg, Germany; hruby.lili2002@gmail.com

**Keywords:** IVF, follicular fluid (ff) mycotoxins, ff steroids (E2; P4), anti-Müllerian hormone (AMH)

## Abstract

The effect of mycotoxin exposure on follicular fluid composition and reproductive outcomes in women undergoing in vitro fertilisation (IVF) was investigated in this study. Twenty-five patients were included, and follicular fluid and serum samples were analysed for various mycotoxins. Principal observations:1. Mycotoxin presence: All examined mycotoxins were detected in follicular fluid. Follicular fluid (ff) levels: Deoxynivalenol (DON), alfa-Zearalenol (α-ZOL), Zearalenone (ZEN), and total aflatoxin (AFs) were significantly higher in follicular fluid than in serum. 2. Follicular fluid and reproductive outcomes: A positive correlation was observed between the ratio of oocytes to total follicles and the follicular Fumonisin B1 (FB1) levels. Multiple linear regression analysis revealed a significant relationship between DON and T-2/HT-2 toxins (T2/HT2) levels in the follicular fluid. 3. Hormone levels: Follicular 17-beta estradiol (E2) and progesterone (P4) levels were higher than the serum levels. Follicular P4 correlated with serum P4 and Anti-Müllerian hormone (AMH) levels. In contrast, follicular E2 did not correlate with plasma E2 levels. 4. Mycotoxin–hormone interactions: A positive correlation was observed between follicular P4 and T2/HT2 toxin levels, whereas a negative correlation was found between ffE2 and ffT2/HT2, and a positive correlation was found between ZEN and E2. Conclusion: This study elucidated the presence of various mycotoxins in the follicular fluid and their potential influence on reproductive outcomes. Further research is warranted to clarify the specific mechanisms underlying these effects and develop strategies for detecting mycotoxin exposure in women undergoing IVF.

## 1. Introduction

Mycotoxins, as secondary metabolites produced by various moulds, can influence the physiological processes of cells, tissues, and organs through several effects. Once inside the cells, they can cause oxidative stress, apoptosis, and direct DNA damage by binding to hormone receptors or the endoplasmic reticulum. Their role as immunomodulators, carcinogens, and endocrine disruptors is known [1,2,3,4]. They can also appear in blood serum, tissues, and other body fluids [5].

The follicular fluid surrounding cumulus cells in tertiary follicles plays a vital role in follicular maturation and the formation of oocytes. Hormones, proteins, free fatty acids, and other factors produced by the theca and granulosa cells all affect the composition of this fluid, and disturbances significantly affect fertility [6].

The follicular fluid primarily comprises blood plasma that filters through the follicle barrier. It also contains proteins, lipids, hormones, and other factors produced by the granulosa and theca cells within the follicle [7,8]. These include growth factors, cytokines, amino acids, proteins, lipids, and polysaccharides. The levels of various substances in the follicular fluid, such as hormones like E2, P4, and 25-hydroxyvitamin D3, as well as fatty acids and noncoding RNAs, are associated with oocyte quality and pregnancy outcome [9,10,11]. Disturbances in this microenvironment can significantly impact oocyte maturation [6,12]. Studies have shown that differences in 17β-estradiol (E2) and progesterone (P4) levels in both serum and follicular fluid can predict variations in oocyte maturation and quality, influencing the success of in vitro fertilisation (IVF) treatment [13,14,15].

There are few data on mycotoxins’ penetration from the serum into the follicular fluid. Based on animal experiments and human in vitro studies, it can be assumed that certain toxins entering the follicular space can trigger inflammatory and oxidative stress processes [16,17] or can disturb the delicate balance of follicular sex steroids by exerting an endocrine disruptor (EDC) effect [13]. Previous studies on animal models have confirmed the presence of some mycotoxins in the follicular fluid and even described a harmful impact on oocyte maturation [18,19]. These studies primarily dealt with the presence and effects of the known EDC, DNA-damaging ZEN, its metabolites, and DON [20]. However, the impact on oocyte development and maturation was also described for ochratoxin-A (OTA) aflatoxin B1 (AFB1) and T2/HT2 toxins in mice and pigs [21]. Aflatoxins can primarily damage oocyte formation and embryonic development by damaging DNA and mitochondrial function, stimulating oxidative stress and apoptotic mechanisms [22,23]. The genotoxicity of ZEN has been confirmed, as it induces DNA fragmentation, apoptosis, and interruption of cell cycle progression [24]. Several studies have shown that ZEN reduces porcine oocyte developmental competence [25].

Similarly, DON directly damages DNA, induces lipid accumulation, and increases inflammation and oxidative stress [26]. DON sensitivity varies across species due to genetic, metabolic, and gut microbiota factors, with humans and pigs particularly vulnerable due to high absorption, prolonged retention, and limited detoxification; tissue-specific accumulation, primarily in the GI tract, liver, kidney, and immune system, and variations in gut microbiota composition further contribute to these differences [27]. OTA induces apoptosis, decreases cell viability, and impairs mouse oocyte maturation and embryonic development [28]. T-2 toxin is considered the most toxic trichothecene mycotoxin and has been shown to exert various toxic effects on farm animals and humans, particularly in tissues with high cell proliferation rates [29]. It has been found to cause significant impairment of the blood–brain barrier function at low nanomolar concentrations [30]. HT-2 toxin, the major metabolite of T-2 toxin, has been less extensively studied but has shown similar toxic effects, albeit at higher concentrations compared to T-2 toxin. For instance, HT-2 toxin exposure disrupted oocyte maturation, induced oxidative stress, and resulted in oocyte apoptosis in mouse studies [29]. In porcine oocytes, HT-2 toxin inhibited polar body extrusion, disrupted meiotic spindle morphology, and affected cytoskeleton structure [31].

Interestingly, the metabolism of HT-2 toxin in the human body has not yet been fully understood. However, human cell line studies have demonstrated that HT-2 toxin can be metabolised to various compounds, and its metabolites can have apoptotic effects in micromolar concentrations [30]. T2 and HT2 toxins causes myriad effects, including inhibiting protein, DNA, and RNA synthesis; oxidative stress [32]; reduced reproduction [33]; and embryo–foetal toxicity [34].

Based on the studies above, mycotoxins may also contribute to reduced folliculogenesis and the development of infertility of unknown origin. However, analyses of the mycotoxin content of human follicular fluids have been lacking until now.

In connection with our study, our goal was to verify the presence of the most frequently occurring mycotoxins in the follicular fluid and to compare the differences between the detectable concentration in the blood plasma and the detected concentration in the follicular fluid. We calculated the correlations between follicle formation and mycotoxin concentrations. The possible effects of mycotoxins on follicular E2 and P4 levels were also investigated.

## 2. Results

Twenty-five follicular fluid samples were analysed. The mean age of the patients was 36.6 (±0.79 SEM) years, and the body mass index (BMI) was 25.3 (±0.73 SEM). The mean AMH value was 2.043 ± 0.46 μg/L (Table 1). Embryo transfer occurred in 13 cases after oocyte retrieval (12 cases on Day 5, 1 on Day 3). In four cases, there were no embryos suitable for implantation; in eight cases, the embryos were frozen (primarily due to too high P4 or thin endometrium).

### 2.1. Serum and Follicular E2 and P4

Higher follicular E2 and P4 levels (*p* < 0.001) were detected compared to serum levels. ffP4 was positively correlated with serum P4 (r = 0.4894, *p* < 0.05, Table 1) and the dosage of used recombinant human FSH (rFSH) treatment (r = 0.4889, *p* < 0.05) and negatively correlated with AMH levels (r = −0.4994, *p* < 0.05). ffE2 did not correlate with plasma E2 levels on day 5 or 12 with rFSH treatment. Interestingly, while a positive correlation was seen between ffP4 and ffT2/HT2 toxin levels (r = 0.46, *p* < 0.05), a moderate negative correlation between ffE2 and ffT2/HT2 was found as well (r = −0.43, *p* < 0.05). ffE2 also positively correlated with BMI (r = 0.50, *p* < 0.05) and ffZEN (r = 0.51, *p* < 0.05). (Supplementary Materials) We found no correlation between the total and dominant follicle number and ffE2 and ffP4 levels.

### 2.2. Impact on Folliculogenesis

**Figure 1:**
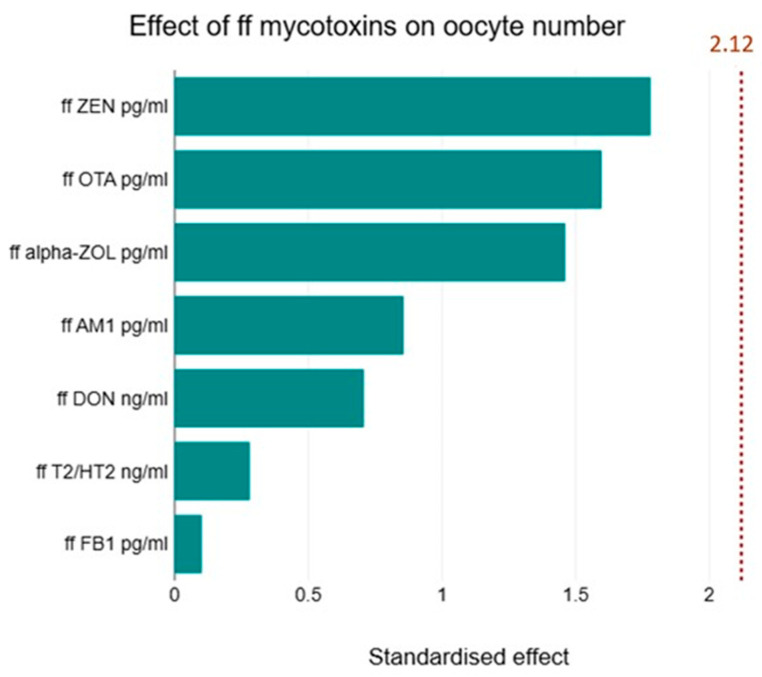
Effect of the level of follicular fluid mycotoxins on oocyte number. Pareto analysis was used to show the possible impact of mycotoxins on oocyte development. The diagram shows the differences that were examined between the impacts of mycotoxins. However, their impact did not reach the level of significance.

Age and AMH were the most decisive factors in the number of follicles detected after the stimulation treatment. In the case of AMH, there was a robust positive correlation (r = 0.77, *p* < 0.0001) with the total number of follicles and a strong negative correlation with age (r = −0.60, *p* < 0.001). A significant positive correlation was detected between the number of follicles formed and the serum E2 serum levels measured on day 12 of the cycle (r = 0.7 *p* < 0.001). However, the follicular E2 levels did not correlate with the total number of follicles or dominant follicles bigger than 15 mm (r = 0.3, *p* = 0.13). Our data did not show any correlation between ffP4 levels and the number of total or dominant follicles. However, we found a significant multiple linear regression between follicular and plasma P4 levels and the ratio of fertilised eggs to the total number of follicles (F = 4.418, *p* < 0.05). We found an interesting positive correlation between the ratio of oocytes to total follicles and follicular FB1 levels (r = 0.6174, *p* < 0.001). This relationship also stood out among the other mycotoxins when multiple linear regression was used (F = 17.61, *p* < 0.001). A Pareto analysis revealed the possible impact of mycotoxins on folliculogenesis (Figure 1).

### 2.3. Serum and Follicular Fluid Mycotoxin Levels

All of the examined toxins were detectable in the follicular fluid. Serum and follicular fluid concentrations were not significantly different for AfM1, OTA, FB1, and T2/HT2 toxins. Substantially higher concentration values were found for DON, α-ZOL, and ZEN in the follicular fluid compared to the serum levels. Interestingly, the serum levels of total aflatoxins were below the limit of detection (LOD) among the tested patients (Table 2).

A positive correlation between serum and follicular fluid levels was detected in the case of AfM1 and ZEN. At the same time, there was no correlation between the serum and follicular fluid concentrations of the other mycotoxins. Among the examined toxins, we found positive and negative correlations in the follicular fluid (Table 3). When multiple linear regression analysis was used, a strong relationship was found between DON and T2/HT2 toxins (F = 6.574, *p* < 0.05) in the follicular fluid. However, no significant relationship was found for the other toxins.

## 3. Discussion

Our study examined the co-presence of six mycotoxins (AF, ZEN, OTA, FB1, DON, and T2/HT2) and two metabolites (α-ZOL and AFM1) in human follicular fluid for the first time. We proved that the measured mycotoxins are present in detectable concentrations in the follicular fluid, even in cases where this value remains below the detection threshold in the blood plasma taken simultaneously. Early follicles take slightly more than 65–70 days to mature [35,36]. The follicular fluid may already appear in the preantral follicles. However, a significant part of the follicular fluid accumulates only ten days before ovulation in the tertiary Graafian follicles [35]. Compared to about 0.03 mL in the early period, the fluid volume can reach up to 2.7 mL in the last days before ovulation [37]. Although mycotoxins in the granulosa and theca cells can disturb the early stages of follicle maturation and hormone secretion [38,39], it is mainly during the last stage of development that toxins taken by food or stored in other tissues and released into the circulation can enter. Their clearance may be limited, or they can even accumulate in the follicular fluid [16]. This hypothesis can be partly supported by the fact that some mycotoxins were not always detectable in the serum taken at the time of follicular fluid aspiration. Still, they were present in most follicular fluid samples. This is especially true in the case of AFs, where none of the samples from the examined patients had detectable amounts of mycotoxins in the serum. Still, their presence was confirmed in the follicular fluid. This can be partly explained by the relatively fast hydroxylation of AFs in the liver and the fact that some mycotoxins, such as AFs, can be retained in the follicular fluid in parallel with the former process [40]. The levels of some mycotoxin metabolites may also differ due to the enzyme activity of granulosa and theca cells [41]. Our results showed no significant difference between blood plasma and follicular fluid levels in the case of AfM1, OTA, FB1, and T2/HT2. Still, at the same time, the concentrations of ZEN, α-ZOL, Afs, and DON were higher in the follicular fluid than in the serum. Human biomonitoring studies have revealed the presence of various mycotoxins in serum and plasma samples across different age groups, highlighting mycotoxins’ widespread exposure. A study comparing infertile and healthy males found that the incidence of multiple mycotoxin exposure was significantly higher in infertile males, with notably increased levels of ochratoxin A, ochratoxin B, and citrinin [42].

Additionally, studies have focused on pregnant women’s ochratoxin A, citrinin, and enniatins in serum collected throughout pregnancy [43]. These studies demonstrated that mycotoxin exposure is prevalent across age groups and populations. The detection of multiple mycotoxins in serum and plasma underscores the importance of comprehensive biomonitoring studies to assess human exposure and potential health risks associated with mycotoxins [44,45].

As a small water-soluble chemical molecule, DON is unlikely to be internalised by endocytosis or pinocytosis. Theoretically, the entry of DON into cells depends on either passive or active transport. It has been elucidated that organic anion transporters (OATs), organic cation transporters (OCTs), and organic anion-transporting peptides (OATPs) are the primary uptake transporters, including OATPs in Caco-2 cells, OATs and OATPs in HepG2 cells, and OATs and OCTs in MDCK cells, respectively [46]. This also suggests that tissue-specific expression of transporters may influence DON absorption in vivo. Bioavailability is a crucial toxicokinetic parameter in assessing toxicosis and susceptibilities in animals. Animals and humans have also demonstrated significant variations in bioavailability following oral administration of DON. Chickens exhibit the lowest bioavailability, with only approximately 15–20% of the toxin absorbed into the circulatory system, whereas rats, pigs, and humans have substantially higher bioavailability (~45–100%) [46]. Mengelers et al. (2019) observed that the administration of DON to human volunteers indicated a bioavailability of 30–98% [47]. One of the reasons may be entrapment due to the binding of human serum albumin; thus, a kind of cumulation may occur [48,49,50,51].

Previous studies have confirmed the presence of these mycotoxins in domestic animals’ follicular fluid [16,52]. Winkler et al. (2015) confirmed similar results of DON concentrations in cows; however, due to differences in human metabolism of ZEN and its metabolites, the α-ZOL pathway is more prominent than β-ZOL in pigs and mice [16,53,54]. The plasma elimination kinetics of DON in humans are similar to those observed in pigs. The half-life (t1/2) in humans was determined to be within the range of 2.9–3.6 h [55]. The follicular presence and effects of DON, ZEN, and α-ZOL on oocyte maturation are well documented; however, in vitro studies and animal experiments have usually investigated the apoptotic effects that damage or delay maturation at a concentration that is an order of magnitude higher than the concentration measured in the human patients [16,56,57,58]. We did not find an apparent effect on folliculogenesis at the concentrations detected in the patients in the present study. This can be partly explained by the small number of cases, the dose-dependent effects of mycotoxins, and the synergistic and sometimes antagonistic effects of their co-presence.

Interestingly, the concentration of FB1 was positively correlated with the ratio of oocytes and follicles. The antioxidant role of FB1 at low concentrations was described by Lolicato et al., where palmitate-induced ROS formation in cumulus cells and follicular fluid and deterioration of mitochondrial function could be suspended with FB1 as a ceramide synthase inhibitor [59]. Nuclear factor erythroid 2-related factor 2 (Nrf2) is a crucial transcription factor that controls the expression of antioxidant enzymes and cytoprotective proteins [60]. It plays a pivotal role in maintaining cellular redox balance and defending against oxidative stress [60,61]. Nrf2 activation occurs in response to various stimuli, including environmental oxidants, resulting in increased antioxidant response elements (AREs) and the initiation of cellular response pathways [61,62]. This effect is even more significant at low levels of oxidative stress. The correlation matrix in Table 2 illustrates the relationship between individual mycotoxin levels. Due to the relatively small number of cases, these values are only indicative, and further tests are necessary to confirm closer correlations.

Numerous reports in the literature indicate the correlation between follicular E2 and P4 levels in animal models and human studies with follicular and oocyte maturation [16,63,64,65]. The follicular E2 and P4 levels were significantly higher than in the serum. ffE2 strongly correlated with BMI and ffZEN, corresponding to previously published results [65]. However, our results did not show a correlation with age, serum E2 levels on days 5 and 12, the number of follicles formed, or the AMH serum level. This may be due to a larger standard deviation of the patients’ BMI values. Similar results regarding the correlation of ffE2 levels were obtained by Lv et al. However, while they found a correlation of ffE2 with the applied gonadotropin treatment, our results did not show such a correlation [66].

The applied rFSH correlated with follicular P4 levels and the ratio of fertilised oocytes to total follicles in the present study. This result is consistent with the conclusions drawn in a previous summary study [65]. Wen X et al. also concluded that egg maturation and fertilisation are unrelated to follicular E2 levels [67]. In our study, we compared the average values of the pool of all extracted follicular fluids with the data on follicular formation.

In contrast, the study mentioned above analysed the individual follicular fluids separately. A slightly lower ffE2 concentration was measured in the mature follicles, which was more significant in follicles with 15 mm diameter than in follicles smaller than 15 mm [67]. Marques-Pizarro et al. described a positive correlation between ffE2 and FSH, LH levels, and serum E2 levels taken during β-human chorionic gonadotropin (βHCG) administration. In contrast, a negative correlation with serum progesterone levels was measured simultaneously. The discrepancy may be partly due to the measurements taken on different cycle days or the fact that only the follicular fluids of more prominent follicles with more than 17 mm diameter were examined [13].

Rare data exist on T2/HT2 toxin occurrence in follicular fluid. In 2014, Jing W et al. reported a dose-dependent inhibition of steroidogenesis by T2 toxin via the cAMP-PKA pathway, as well as stimulation of apoptosis-induced Caspase 3 or oxidative stress (in the μM range) in the granulosa cells (1–100 nM) [68,69]. This inhibitory effect can be concentration-dependent since granulosa cells’ progesterone production can vary in concentration, and it is an IGF-I-mediated process [70]. Caloni F et al. reached a similar result regarding the production of E2 and P4 in granulosa cells, where compared to E2, the stimulation and inhibition of P4 production ranged between even narrower concentration limits [71]. The positive correlation between follicular E2, ZEN, and α-ZOL levels suggests an exciting connection. A similar relationship and elevated E2 levels were described in the presence of ZEN and α-ZOL in bovine granulosa cells, although at orders of magnitude higher concentrations [72]. α-ZOL metabolism may be a defence mechanism to compensate for the estrogenic effects of ZEN [73].

The presence of mycotoxins in follicular fluid underscores the need for a comprehensive approach to food safety and environmental health. It emphasises the importance of monitoring and controlling low-level mycotoxin contamination in the food chain, as well as understanding the potential long-term effects of chronic, low-dose exposure to these toxins on reproductive health [74]. Further studies should focus on developing more sensitive biomarkers and conducting epidemiological studies to better understand the relationship between mycotoxin exposure and reproductive outcomes, as well as implementing strategies to mitigate exposure risk.

## 4. Conclusions

In conclusion, this study represents the first demonstration of various mycotoxins in human follicular fluid and elucidates the relationship between mycotoxins and reproductive parameters. However, the sample size is sufficient to demonstrate trends, but a larger cohort would be necessary to strengthen the findings. The study’s findings revealed that several mycotoxins were detectable in the follicular fluid, with some instances showing higher concentrations than in serum. This observation suggests that follicles can accumulate ZEN and its metabolite alpha-ZOL, AFs, and total DON (potentially due to HSA-binding). The observed correlations between mycotoxins and levels of hormones (E2, P4) detectable in follicular fluid indicate that mycotoxins may influence follicle maturation and hormone production.

## 5. Materials and Methods

Infertile patients who underwent controlled rFSH stimulation treatment were included. All patients lived in Hungary’s capital city agglomeration. The climate in Hungary is temperate continental, with warm summers and cold winters. This climate can be conducive to the growth of certain fungi that produce mycotoxins, particularly during periods of high humidity and warm temperatures. Some of the most common mycotoxins found in Hungarian crops include deoxynivalenol (DON), Aflatoxins, Ochratoxin A (OTA), Fumonisins, and T2- HT2 toxins, and they could appear in animal and dairy products as well. The patients included in the study were treated, and oocytes were extracted in June 2024. After obtaining detailed verbal and written information, 25 patients were included in the study after signing a consent form approved by the regional ethics committee (SE-RKEB 86/2023). During the cycle, serum hormone tests were performed on days 5 and 12 (E2, FSH, LH, AMH). Before the egg retrieval, the visible follicles were accurately measured during a transvaginal ultrasound examination. Oocyte retrievals were performed at Central of Assisted Reproduction, Semmelweis University, Budapest. On the day of oocyte retrieval, blood was retaken to determine serum mycotoxin levels. After that, the follicles were aspirated, and the oocytes were extracted under surgical conditions. After the isolation of the oocytes, a sample was taken from the remaining follicular fluid and centrifuged. Cases where the follicular fluid was visibly contaminated with blood were excluded. The follicular fluid was collected as a pool obtained from all follicular fluids, since separate collection significantly increases the risk of intervention and contamination with blood [16]. Serum and follicular samples were stored at −70 °C.

### 5.1. Experimental Setup on Samples

Afs (M1, B1, B2, G1, G2), DON, FB1, OTA, ZEN, α-ZOL, T2/HT2 toxins, estradiol, progesterone, and AMH levels were quantified in blood plasma and follicular fluid samples obtained according to the methodology delineated below.

### 5.2. Serum and ff Steroid Analyses

For sex steroid analyses in plasma and follicular fluid, 17-beta-estradiol (Cat No: DNOV003, NovaTec Immundiagnostica, Dietzenbach, Germany) and progesterone (Cat No: DNOV006, NovaTec, Dietzenbach, Germany) kits were utilised. The immunoassay was conducted in accordance with the manufacturer’s instructions, and the serum and ff samples were measured in triplicate [75,76].

Measurements were acquired, and data were analysed using a Thermo MultiskanTM FC microplate reader (Waltham, MA, USA) equipped with SkanIt RE software (version 6.1.1.7). The absorbance was measured at 450 nm with a reference wavelength of 630 nm.

### 5.3. Serum Anti-Müllerian Hormone (AMH) Measurements

AMH was determined using the Beckman Coulter Access 2 Immunoassay System (Brea, California USA) using the chemiluminescence immunoassay (CLIA) method. The Access AMH Advanced assay (Cat. No: B13127) is a paramagnetic particle chemiluminescent immunoassay for quantitatively determining anti-Müllerian hormone levels in human serum utilising the Access Immunoassay Systems. The measurements were conducted in duplicate according to the manufacturer’s instructions.

### 5.4. Serum FSH and LH Measurements

FSH and LH levels in serum were determined using an Atellica IM 1600 immunoassay analyser with chemiluminescence testing methodology (CLIA) using advanced acridinium ester technology (Siemens Healthineers, Cary, NC, USA).

### 5.5. Mycotoxin Analyses

Mycotoxin measurements were performed on follicular fluid and plasma samples. The assays were performed using ELISA optimisation for the serum and tissue samples [75,77].

ZEN, OTA, FB1, DON, T2/HT2 toxin, Afs (total aflatoxins: B1, B2, G1, and G2), and aflatoxin M1 were analysed using immunoassays. In the case of α-ZOL, a GC-MS measurement was applied.

ZEN analyses used commercial Ridascreen Zearalenone (Art No.: R1401 R-Biopharm, Arnhem, Germany) enzyme immunoassay kits. The serum and FF sample preparation RIDA© C18 column (art No: R2002, R-Biopharm, Arnhem, Germany) was used according to the manufacturer’s instructions, and the samples were measured in triplicate.

The blood serum and ff sample preparation and extraction for α-ZOL were the same as those for ZEN. As reported, ZEN metabolite α-ZOL in blood was tested in triplicate by gas chromatography with a mass spectrometer (GC-MS) [75].

Serum and follicular fluid (ff) samples for ochratoxin A (OTA) analysis were thawed, subsequently diluted/extracted with a threefold acetonitrile/water solution (84/16, *v*/*v*), and subjected to agitation in an orbital shaker for 15 min at room temperature. The resulting extracts were centrifuged at ambient temperature at 8000× *g* for 5 min; the supernatants were collected and diluted with 0.01 M phosphate-buffered saline (PBS), pH 7.4. OTA serum analyses were quantified utilising the previously reported HPLC-FLD method [77,78]. FF recovery was 87.65%.

Fumonisin B1 (FB1) was quantified utilising the EUROPROXIMA Fumonisin (5121FUM) (R-Biopharm, Arnhem, Germany) assay kit. This commercial kit was validated for serum and various animal tissues. As previously reported [75], prior to the analysis of the FF samples, the recovery rate of FB1 spiked tissue was evaluated, which was 72.15%. Analyses were conducted in accordance with the manufacturer’s instructions. Samples were measured in triplicate.

T2/HT2-Toxin analyses were carried out according to the manufacturer’s instructions. Bio-Shield T2/HT-2 (Prognosis Biotech, Larissa, Greek) ELISA kits were used to determine T2/HT2 toxins. The blood serum and FF sample preparation for the T2/HT-2 toxins on the C18 column were the same as those for ZEN. Samples were measured in triplicate. Following evaporation, the dried residue was re-dissolved in 500 µL of methanol–water (35:65 *v*/*v*%), as previously reported by Unicsovics et al., 2024 [75].

Total aflatoxins (B1, B2, G1, G2), TW AfM1, and DON contents were determined using Toxi-Watch (Soft Flow Ltd., Pécs, Hungary) ELISA kits, previously validated for serum samples and different organs/tissues according to the manufacturer’s instructions. Ff recovery rates for AfB1 were 72.15%, for AfM1 87.25% and for DON 70.26%.

### 5.6. Statistical Analysis

The statistical analyses were conducted utilising GraphPad Prism software (Version 10.3.1. 2024, GraphPad, La Jolla, CA, USA). Continuous variables were compared using an independent t-test or Mann–Whitney test, based on the Shapiro–Wilk normality test. Linear regression was employed to analyse the independent correlated factors; Pearson or non-parametric Spearman correlation was utilised to determine the relationship between values. *p* < 0.05 was deemed to indicate statistical significance.

## Figures and Tables

**Table 1:** Biophysical, hormonal, and follicular maturation parameters. The table contains the mean, median, standard deviation, and minimum and maximum levels or numbers of the main biophysical and hormonal parameters. Descriptive statistics on folliculogenesis, oocytes, and useable embryos were also included. * The follicular level is significantly higher compared to serum. ^†^ The serum and follicular levels correlate. Mann–Whitney nonparametric u-test was used.

	Mean	Standard Deviation	Median	Minimum	Maximum
					
Age	35.9	4.0	36.0	28	44
BMI	25.3	4.5	24.3	18.4	34.7
Anti-Mullerian hormone (μg/L)	2.043	0.46	2.277	0.15	8.38
Total number of follicles (n)	10.7	6.4	10	2	27
Dominant follicles (Ø > 15 mm, n)	8.1	5.1	7	1	21
Useable embryos (n)	1.9	2.2	1	0	9
Ratio of oocytes/all follicles	0.41	0.41	0.4	0	1.17
Ratio of embryos/all follicles	0.13	0.13	0.09	0	0.6
Ratio of embryos/dominant follicles	0.18	0.2	0.13	0	0.9
Serum P4 (μg/L)	1.57 ^†^	1.06	1.2	0.4	5.1
ff P4 (μg/L)	5170.22 *^,†^	1991.96	5112.4	1166.6	9110.9
Serum E2 5.day (pg/mL)	43.5	15.3	46.6	17.1	66
Serum E2 12.day (pg/mL)	1995.66	1237.04	1598.6	477	4575
ff E2 (pg/mL)	36,591.94 *	3743.42	37,261.6	29,180.6	41,583.1
Serum FSH (IU/L)	7.04	2.7	6.1	3	14.1
Serum LH (IU/L)	4.62	1.7	4.7	1.4	8.1

**Table 2:** Serum and follicular mycotoxin levels. The table contains the mean, median, standard deviation, and minimum and maximum mycotoxin level concentrations in the serum and follicular fluid. * The follicular mycotoxin level was significantly higher compared to serum. ^†^ The serum and follicular levels correlated. Mann–Whitney nonparametric u-test was used.

	Mean	Standard Deviation	Median	Median in nM/mL	Minimum	Maximum
						
serum Aflatoxin (pg/mL)	0	0	0	0	0	0
ff Aflatoxin (pg/mL)	27.55	60.62	0	0	0	270
serum Aflatoxin M1 (pg/mL)	6.24 ^†^	11.46	0	0	0	43.33
ff Aflatoxin M1 (pg/mL)	9.16 ^†^	13.3	6.17	1.97 × 10^−5^	0	58.81
serum Zearalenone (pg/mL)	81.34 ^†^	100.58	58.14	1.82 × 10^−4^	0	391.19
ff Zearalenone (pg/mL)	241.93 *^,†^	179.34	191.67	6.01 × 10^−4^	0	756.37
serum alpha-Zearalenol (pg/mL)	192.52	121.72	212	6.34 × 10^−4^	0	354
ff alpha-Zearalenol (pg/mL)	554.56 *	194.18	550	1.68 × 10^−3^	228	926
serum Ochratoxin A (pg/mL)	7.44	6.15	6	1.48 × 10^−5^	0	22
ff Ochratoxin A (pg/mL)	6.56	5.9	8	1.98 × 10^−5^	0	16
serum Fumonisin B1 (pg/mL)	110.75	120.96	82.6	1.48 × 10^−4^	0	388.4
ff Fumonisin B1 (pg/mL)	68.8	83.17	40	7.22 × 10^−5^	0	272
serum Deoxynivalenol (ng/mL)	1.58	1.49	1.16	3.91 × 10^−3^	0	4.36
ff Deoxynivalenol (ng/mL)	5.11 *	1.66	5.06	1.71 × 10^−2^	2.14	8.78
serum T2/HT2 toxin (ng/mL)	1.73	1.39	1.89	3.64 × 10^−3^	0	4.25
ff T2/HT2 toxin (ng/mL)	0.97	1.34	0.36	6.94 × 10^−4^	0	4.69

**Table 3:** Correlation between mycotoxin levels in follicular fluid. Our study found a positive correlation between ffDON and ffT2/HT2 and between ffDON and ffAfM1. There was a positive correlation between ffα-ZOL and ffT2/HT2 toxin. A negative correlation was seen between ffα-ZOL and ffOTA. * stands for significant correlation between mycotoxin concentrations in the follicular fluid.

		ff AfM1 pg/mL	ff ZEN pg/mL	ff OTA pg/mL	ff T2/HT2 ng/mL	ff FB1 pg/mL	ff α-ZOL pg/mL	ff DON ng/mL
ff AfM1 pg/mL	Correlation	1	−0.26	−0.15	0.25	0.32	−0.12	0.41
	***p*** **-value**		**0.212**	**0.477**	**0.24**	**0.133**	**0.569**	**0.036** *
ff ZEN pg/mL	Correlation	−0.26	1	0.22	−0.21	−0.3	0.16	0.03
	***p*** **-value**	**0.212**		**0.297**	**0.324**	**0.161**	**0.442**	**0.873**
ff OTA pg/mL	Correlation	−0.15	0.22	1	−0.4	0.04	−0.41	−0.29
	***p*** **-value**	**0.477**	**0.297**		**0.051**	**0.846**	**0.045** *	**0.173**
ff T2/HT2 ng/mL	Correlation	0.25	−0.21	−0.4	1	0.3	0.49	0.46
	***p*** **-value**	**0.24**	**0.324**	**0.051**		**0.149**	**0.016** *	**0.023** *
ff FB1 pg/mL	Correlation	0.32	−0.3	0.04	0.3	1	−0.18	0.06
	***p*** **-value**	**0.133**	**0.161**	**0.846**	**0.149**		**0.407**	**0.774**
ff alpha-ZOL pg/mL	Correlation	−0.12	0.16	−0.41	0.49	−0.18	1	0.36
	***p*** **-value**	**0.569**	**0.442**	**0.045** *	**0.016** *	**0.407**		**0.089**
ff DON ng/mL	Correlation	0.41	0.03	−0.29	0.46	0.06	0.36	1
	***p*** **-value**	**0.036** *	**0.873**	**0.173**	**0.023** *	**0.774**	**0.089**	

## Data Availability

The original contributions presented in the study are included in the article/Supplementary Materials, further inquiries can be directed to the corresponding authors.

## Supplementary Materials

The following supporting information can be downloaded at: https://www.mdpi.com/article/10.3390/toxins16120509/s1, Figure S1: Spearman correlation analysis between ff 17-beta-oestradiol and ff ZEN content; Figure S2: Spearman correlation analysis between ff 17-beta-oestradiol and ff alpha-ZOL content; Figure S3: Spearman correlation analysis between ff progesterone and ff ZEN content; Figure S4: Spearman correlation analysis between ff progesterone and ff alpha-ZOL content; Figure S5: Mycotoxin co-occurrence in serum samples; Figure S6: Mycotoxin co-occurrence in follicular fluid samples; Table S1: Correlation matrix; Table S2: Serum hormone levels.
