# Supplementary material for: The Potential Influence of the Presence of Mycotoxins in Human Follicular Fluid on Reproductive Outcomes
Source: Toxins (Basel). 2024 Nov 25;16(12):509. doi: 10.3390/toxins16120509 (PMC11728479; doi:10.3390/toxins16120509)
Supplement: Supplementary file 1 [file toxins-16-00509-s001.zip › toxins-3308988-supplementary.pdf]

Supplementary material

The Potential Influence of the Presence of Mycotoxins in Human Follicular Fluid on Reproductive Outcomes

Apolka Szentirmay, Zsófia Molnár, Patrik Plank, Miklós Mézes, Attila Sajgó, Attila Martonos, Tímea Buzder, Miklós Sipos , Lili Hruby, Zsuzsanna Szőke and Levente Sára

Figure S1. Spearman correlation analysis between ff 17-beta-oestradiol and ff ZEN content.

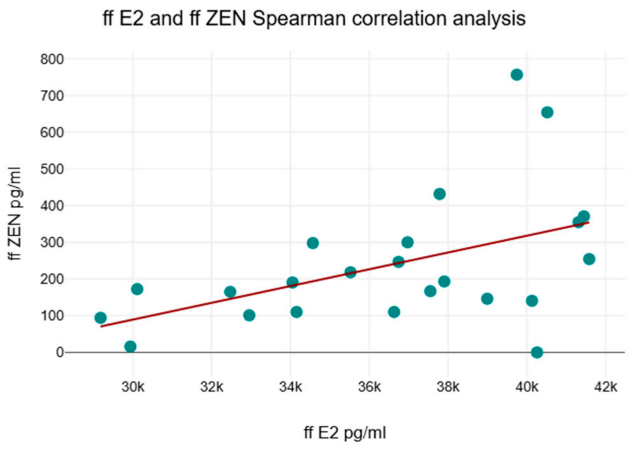

	r	p
ff E2 pg/ml and ff ZEN pg/ml	0.51	0.012

Figure S2. Spearman correlation analysis between ff 17-beta-oestradiol and ff alpha-ZOL content.

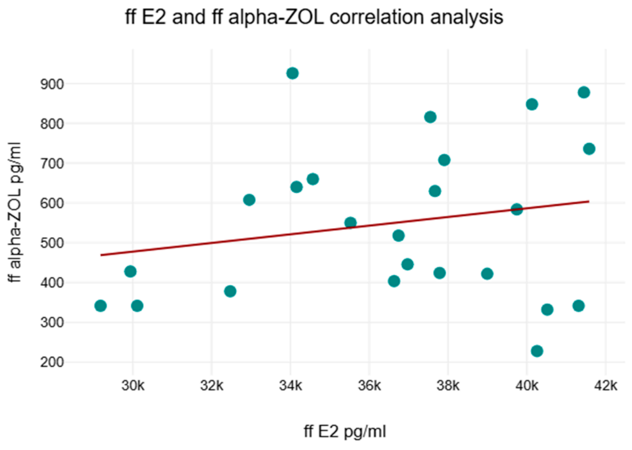

	r	p
ff E2 pg/ml and ff alpha-ZOL pg/ml	0.13	0.56

Figure S3. Spearman correlation analysis between ff progesterone and ff ZEN content.

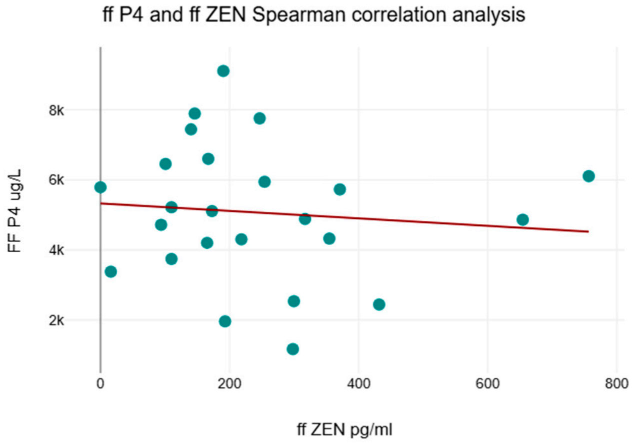

	r	p
ff ZEN pg/ml and FF P4 ug/L	−0.15	0.475

Figure S4. Spearman correlation analysis between ff progesterone and ff alpha-ZOL content.

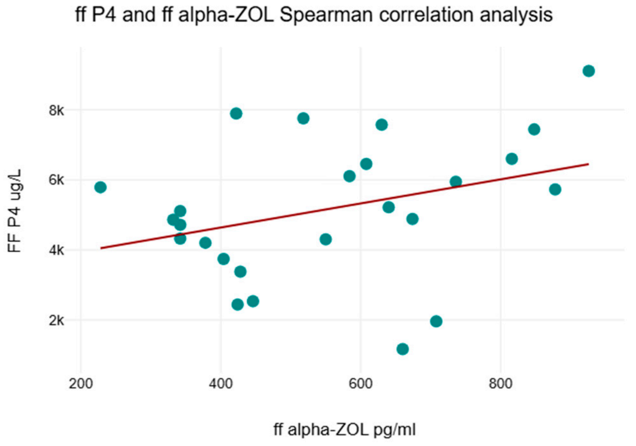

	r	p
ff alpha-ZOL pg/ml and FF P4 ug/L	0.3	0.138

Figure S5. Mycotoxin co-occurence in serum samples.

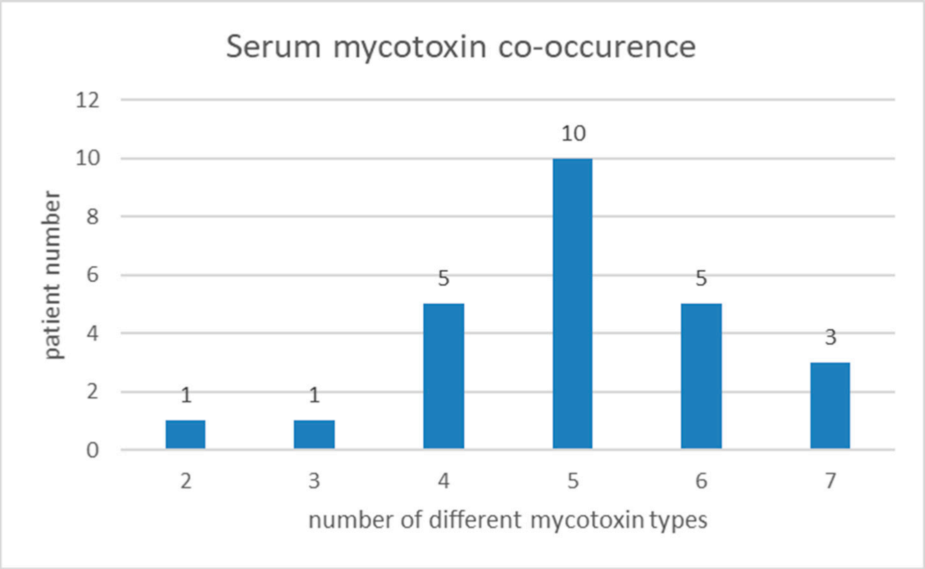

Figure S6. Mycotoxin co-occurence in follicular fluid samples.

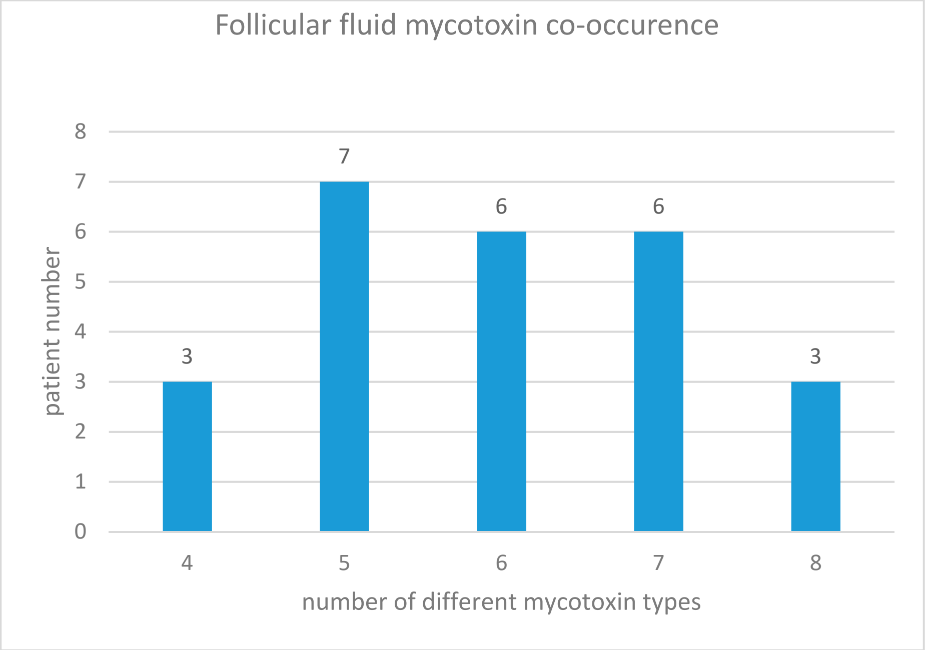

Table S1. Correlation matrix.

		ff E2 pg/ml	serum E2 Day5. pg/ml	serum E2 Day 12. pg/ml	serum P4 ug/L	FF P4 ug/L	serum LH IU/L	serum FSH IU/L	Total usable embryo number	Female age	total follicle number	Follicles < 15mm fleet	ratio of embryo number to total follicles	ratio of embryo number to 15mm < follicles	ratio of oocytes to total follicles
ff E2 pg/ml	Correlation	1	-0.29	0.2	-0.16	0.15	-0.17	-0.18	-0.17	-0.13	0.24	0.25	-0.36	-0.44	-0.01
	**p**		**0.223**	**0.394**	**0.51**	**0.523**	**0.467**	**0.45**	**0.462**	**0.592**	**0.308**	**0.281**	**0.116**	**0.052**	**0.954**
serum E2 Day5. pg/ml	Correlation	-0.29	1	-0.08	-0.19	-0.15	0.49	0.32	0.11	0.14	-0.28	-0.14	0.21	0.13	-0.12
	**p**	**0.223**		**0.741**	**0.42**	**0.516**	**0.028***	**0.17**	**0.654**	**0.548**	**0.24**	**0.565**	**0.382**	**0.577**	**0.619**
serum E2 Day 12. pg/ml	Correlation	0.2	-0.08	1	0.62	0.35	-0.1	-0.42	0.05	0.12	0.6	0.78	-0.25	-0.38	-0.37
	**p**	**0.394**	**0.741**		**0.003***	**0.132**	**0.674**	**0.064**	**0.819**	**0.612**	**0.005***	**<.001****	**0.298**	**0.097**	**0.112**
serum P4 ug/L	Correlation	-0.16	-0.19	0.62	1	0.49	-0.05	-0.48	0.14	0.22	0.4	0.62	-0.11	-0.16	-0.09
	**p**	**0.51**	**0.42**	**0.003***		**0.018***	**0.834**	**0.034***	**0.57**	**0.353**	**0.079**	**0.003****	**0.632**	**0.513**	**0.705**
FF P4 ug/L	Correlation	0.15	-0.15	0.35	0.49	1	-0.21	-0.32	0.22	0.47	0.12	0.19	0.11	0.03	0.01
	**p**	**0.523**	**0.516**	**0.132**	**0.018***		**0.379**	**0.166**	**0.359**	**0.037***	**0.605**	**0.412**	**0.642**	**0.901**	**0.982**
serum LH IU/L	Correlation	-0.17	0.49	-0.1	-0.05	-0.21	1	0.44	0.2	-0.42	-0.21	0.01	0.23	0.18	-0.1
	**p**	**0.467**	**0.028***	**0.674**	**0.834**	**0.379**		**0.05***	**0.397**	**0.063**	**0.378**	**0.963**	**0.323**	**0.448**	**0.664**
serum FSH IU/L	Correlation	-0.18	0.32	-0.42	-0.48	-0.32	0.44	1	0.2	-0.21	-0.37	-0.34	0.35	0.38	0.29
	**p**	**0.45**	**0.17**	**0.064**	**0.034***	**0.166**	**0.05***		**0.395**	**0.371**	**0.104**	**0.147**	**0.126**	**0.098**	**0.217**
Total usable embryo number	Correlation	-0.17	0.11	0.05	0.14	0.22	0.2	0.2	1	0.05	0.06	0.12	0.82	0.78	0.08
	**p**	**0.462**	**0.654**	**0.819**	**0.57**	**0.359**	**0.397**	**0.395**		**0.824**	**0.795**	**0.603**	**<.001****	**<.001****	**0.732**
Female age	Correlation	-0.13	0.14	0.12	0.22	0.47	-0.42	-0.21	0.05	1	-0.1	-0.02	0.04	0.02	0.03
	**p**	**0.592**	**0.548**	**0.612**	**0.353**	**0.037***	**0.063**	**0.371**	**0.824**		**0.668**	**0.948**	**0.871**	**0.926**	**0.904**
total follicle number	Correlation	0.24	-0.28	0.6	0.4	0.12	-0.21	-0.37	0.06	-0.1	1	0.83	-0.42	-0.41	-0.3
	**p**	**0.308**	**0.24**	**0.005***	**0.079**	**0.605**	**0.378**	**0.104**	**0.795**	**0.668**		**<.001****	**0.063**	**0.072**	**0.205**
Follicles < 15mm fleet	Correlation	0.25	-0.14	0.78	0.62	0.19	0.01	-0.34	0.12	-0.02	0.83	1	-0.3	-0.45	-0.37
	**p**	**0.281**	**0.565**	**<.001****	**0.003***	**0.412**	**0.963**	**0.147**	**0.603**	**0.948**	**<.001****		**0.207**	**0.047***	**0.105**

ratio of embryo number to total follicles	Correlation	-0.36	0.21	-0.25	-0.11	0.11	0.23	0.35	0.82	0.04	-0.42	-0.3	1	0.93	0.13
	p	0.116	0.382	0.298	0.632	0.642	0.323	0.126	<.001 **	0.871	0.063	0.207		<.001 **	0.572
ratio of embryo number to 15mm< follicles	Correlation	-0.44	0.13	-0.38	-0.16	0.03	0.18	0.38	0.78	0.02	-0.41	-0.45	0.93	1	0.3
	p	0.052	0.577	0.097	0.513	0.901	0.448	0.098	<.001 **	0.926	0.072	0.047 *	<.001 **		0.197
ratio of oocytes to total follicles	Correlation	-0.01	-0.12	-0.37	-0.09	0.01	-0.1	0.29	0.08	0.03	-0.3	-0.37	0.13	0.3	1
	p	0.954	0.619	0.112	0.705	0.982	0.664	0.217	0.732	0.904	0.205	0.105	0.572	0.197	

Table S2. Serum hormones levels.

	serum P4 µg/L	serum E2 Day5 pg/mL	serum E2 Day12 pg/mL	serum LH IU/L	serum FSH IU/L
Mean	1.57	43.5	1995.66	4.62	7.04
Median	1.2	46.6	1598.6	4.7	6.1
Std. Deviation	1.06	15.3	1237.04	1.7	2.7
Minimum	0.4	17.1	477	1.4	3
Maximum	5.1	66	4575	8.1	14.1
